# Current Situation of Medication Adherence in Hypertension

**DOI:** 10.3389/fphar.2017.00100

**Published:** 2017-03-01

**Authors:** Bernard Vrijens, Sotiris Antoniou, Michel Burnier, Alejandro de la Sierra, Massimo Volpe

**Affiliations:** ^1^WestRock HealthcareVisé, Belgium; ^2^Department of Public Health, University of LiègeLiège, Belgium; ^3^Barts Health NHS TrustLondon, UK; ^4^Department of Nephrology and Hypertension, University Hospital LausanneLausanne, Switzerland; ^5^Internal Medicine Department, Hospital Mutua Terrassa, University of BarcelonaBarcelona, Spain; ^6^Department of Clinical and Molecular Medicine, Faculty of Medicine and Psychology, University of Rome “La Sapienza”Rome, Italy; ^7^IRCCS NeuromedPozzilli, Italy

**Keywords:** adherence, antihypertensive, initiation, implementation, medication, persistence

## Abstract

Despite increased awareness, poor adherence to treatments for chronic diseases remains a global problem. Adherence issues are common in patients taking antihypertensive therapy and associated with increased risks of coronary and cerebrovascular events. Whilst there has been a gradual trend toward improved control of hypertension, the number of patients with blood pressure values above goal has remained constant. This has both personal and economic consequences. Medication adherence is a multifaceted issue and consists of three components: initiation, implementation, and persistence. A combination of methods is recommended to measure adherence, with electronic monitoring and drug measurement being the most accurate. Pill burden, resulting from free combinations of blood pressure lowering treatments, makes the daily routine of medication taking complex, which can be a barrier to optimal adherence. Single-pill fixed-dose combinations simplify the habit of medication taking and improve medication adherence. Re-packing of medication is also being utilized as a method of improving adherence. This paper presents the outcomes of discussions by a European group of experts on the current situation of medication adherence in hypertension.

## Introduction

Poor adherence to treatments for chronic diseases is a worldwide problem and was highlighted as a problem of striking magnitude by the World Health Organization [WHO] (2003). Adherence is of particular concern in hypertension, with about half of the patients prescribed an antihypertensive drug stopping taking it within 1 year, in a longitudinal study of electronically compiled dosing histories of 4783 patients (Vrijens et al., 2008). While, it is recognized that awareness of adherence has increased in recent years, there is still a long way to go.

**FIGURE 1:**
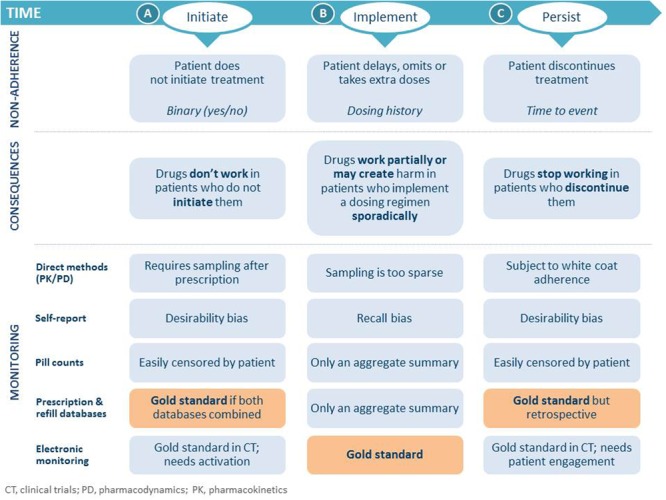
**Process of medication adherence, non-adherence, its consequences and methods of monitoring (Vrijens et al., 2012; Vrijens and Heidbuchel, 2015)**. Permission granted by Oxford University Press.

Adherence is key to therapeutic success; however, it is a multifaceted issue and should not be considered as a dichotomous variable (adherent versus non-adherent). Interestingly, drug adherence goes beyond pill consumption and is a reflection of healthy behavior (Simpson et al., 2006). Medication adherence can be defined as the process by which patients takes their medications as prescribed (Vrijens et al., 2012) and is a dynamic process that changes over time. Adherence consists of three components, which need to be considered separately: (A) initiation, (B) implementation, and (C) persistence (**Figure 1**; Vrijens et al., 2012). The concept of percentage adherence is misleading as it does not reflect these three components (Vrijens, 2016). Persistence with antihypertensive treatment significantly reduces long-term cardiovascular risk (Corrao et al., 2011).

Non-adherence occurs when a patient does not: initiate a new prescription, implement as prescribed, or persist with treatment (**Figure 1**). Failure of patients to fill prescriptions when new medications are started has been shown to be as high as 28% in an analysis of 195,930 electronic prescriptions (Fischer et al., 2010). Suboptimal daily implementation of the prescribed regimen was one of the most common factors for poor adherence with once daily antihypertensive treatment in a longitudinal database study (Vrijens et al., 2008). On any day about 10% of the patients omitted their scheduled dose. In a cohort of 16,907 patients prescribed oral medications for one of a variety of medical conditions in 95 studies, almost 40% of participants had discontinued treatment by 1 year, and 4% never initiated treatment (Blaschke et al., 2012). The consequences of medication non-adherence are drugs not or stopping working, or working partially or creating harm and stopping working (**Figure 1**). Variable adherence also creates drug-specific issues of periodic loss of effectiveness, occasional toxicity, and eventually apparent drug resistance (Blaschke et al., 2012). There is a need to understand the concept of a drug’s “forgiveness” in order to improve understanding of adherence in the future. A drug’s “forgiveness” is best described as the post-dose duration of action minus the dosing frequency (Osterberg et al., 2010).

**FIGURE 2:**
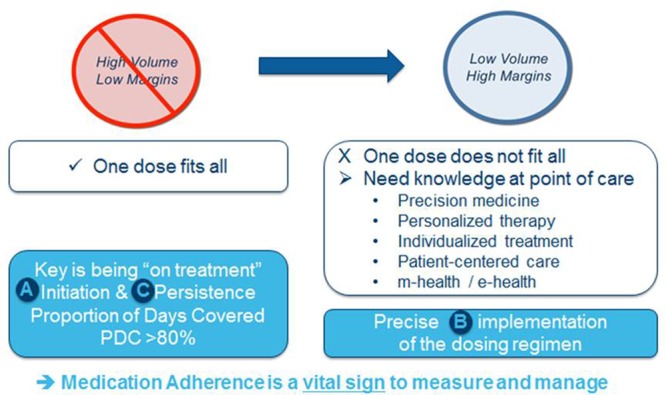
**The changing pharma model (adapted from Personal Communication: Tufts Centre for the Study of Drug Development, 2012)**.

Persistence and initiation are better in clinical trials than in clinical practice; however, implementation is more of a patient attribute and there is no difference in either setting. The pharma model is changing from one dose fits all toward personalized, precision and individualized medicine (Personal Communication: Tufts Centre for the Study of Drug Development, 2012), with adherence being a vital sign to measure and manage (**Figure 2**). Adherence should also be incorporated as a measure in drug development studies (phase II, III, and IV) in line with how drug-related adverse events are currently recorded including discontinuation (Vrijens and Urquhart, 2014). There is also a unique opportunity to improve adherence at initiation of treatment and for approximately 1 week after, and then at treatment failure before escalating therapy. For example, a survey of data collected from 23 community pharmacies in south east England reported that 30% (67/226) of patients still taking a new medication at 10 days were non-adherent (Barber et al., 2004). Problems caused by medicines were categorized as: side effects, difficulties with the practical aspects of taking the medication and necessity concerns. This has led to an initiative by which patients are encouraged to visit their community pharmacist in order to support adherence.

A group of European clinicians and a biostatistician recently met to discuss the current situation of medication adherence, its economic consequences, management, and strategies to improve adherence. This paper presents the outcome of those discussions with particular reference to adherence to antihypertensive medication.

## Economic Consequences of Non-Adherence

The costs of non-adherence to medication are both personal and economic, with knock-on costs as a result of increased demands for healthcare resources if there is deterioration in patients’ health (NICE, 2009). Lack of medication adherence is estimated to cost European governments €125 billion per year; and cost arising due to complications of poor adherence represents 14% of total healthcare expenditure in the United Kingdom’s National Health Service (EFPIA, 2013). It is recognized that payment for drug treatment has some impact on drug adherence but doesn’t prevent non-adherence. In the US, the relationship between non-adherence and associated costs has been depicted as a continuous cycle, with poor medication adherence leading to poor health outcomes, increased service utilization and health care costs, which are passed on to the patient and then lead to further effects on adherence (Iuga and McGuire, 2014).

The IMS Institute for Healthcare Informatics, using a global modeling approach, identified a $500 billion (€455 billion) saving across 186 countries with the responsible use of medicines (IMS, 2012). Responsible use of medicines implies that “activities, capabilities, and existing resources of health system stakeholders are aligned to ensure patients receive the right medicines at the right time, use them appropriately, and benefit from them” (IMS, 2012). About 8% of the global total health expenditure, could be avoided from adherence to medicine (IMS, 2012).

**FIGURE 3:**
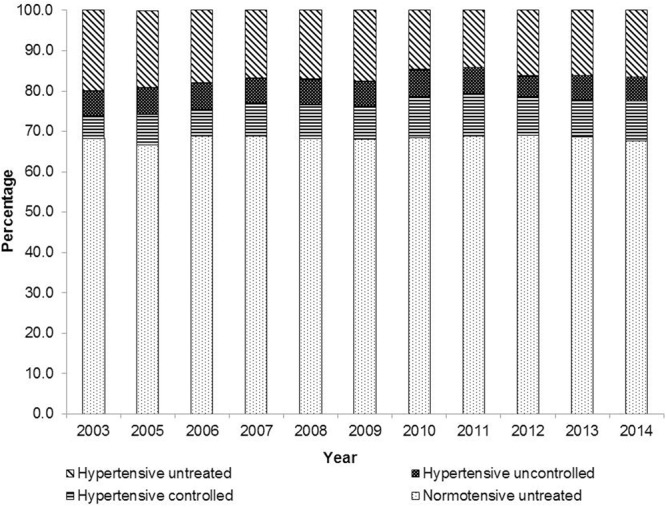
**Prevalence of hypertension and blood pressure control in UK between 2003 and 2014 (Health Social Care Information Centre, 2015).** Blood pressure was not measured in 2004.

Whilst a gradual trend to improve treatment of hypertension has been seen in the UK between 2003 and 2011, the percentage of patients who are hypertensive and uncontrolled has remained consistent (**Figure 3**; Health Social Care Information Centre, 2015). In the UK the annual cost of medicine wastage in primary care is estimated to be £300 million (€333 million), with £100–150 million (€111–166.50 million) identified as avoidable, according to research by York Health Economics Consortium, The School of Pharmacy, University of London (2010). The research also evaluated the cost of non-adherence in six long-term conditions, including hypertension. Savings of just over £100 million (€111 million) per year could be achieved if 80% of patients with hypertension were adherent with treatment (York Health Economics Consortium, The School of Pharmacy, University of London (2010)).

The costs related to hypertension and the economic impact of increasing adherence to antihypertensive therapy have been investigated in five European countries (Italy, Germany, France, Spain, and England), using a probabilistic prevalence-based model, over a 10-year period (Mennini et al., 2015). This model indicated that a total saving of €332 million could be achieved by increasing adherence to antihypertensive therapy to 70%. Studies such as this can help inform decision makers and aid understanding of the importance of adherence. It is important to note that the adherence goal of ≥80% originally came from pharmacy refill claims databases and is not necessarily valid in all clinical situations, for example resistant hypertension, and does not address the drug’s forgiveness as described earlier.

Hypertension is a serious public health issue in low- to middle-income countries (Nielsen et al., 2017), and affordability of medication is an important consideration as this is a problem for medication adherence (Choudhry et al., 2016). A recent systematic review of the literature on non-adherence to antihypertensive medication, among adults in low- and middle-income countries, has highlighted that this is more problematic in some parts of the world (Nielsen et al., 2017). Affordability affects the treatment initiation and persistence components of adherence, as patients who cannot afford the medications typically do not buy them.

Treatment escalation is one of the drivers for increased cost: poor adherence leads to treatment failure, disease progression and more complex treatments, which then lead on to further impact adherence. Adherence is perceived by payers to be associated with increased costs, and there is a need to raise awareness that reimbursement to avoid treatment escalation is beneficial, e.g., supporting ambulatory blood pressure monitoring. Furthermore, most of the estimates of non-adherence are top down and are not sequential in terms of time. However, the key message remains that the number of patients who are non-adherent is high and this jeopardizes the healthcare budget.

## Management of Adherence

Low adherence is the most common cause of apparent resistant hypertension (Jung et al., 2013). Poor adherence to antihypertensive therapy is associated with increased risks of coronary and cerebrovascular events (Corrao et al., 2011). In terms of the management of adherence the objective is to achieve the best use, by patients, of appropriately prescribed medicines in order to maximize the potential for benefit and minimize the risk of harm (Vrijens et al., 2012). The European Society of Hypertension (ESH)/European Society of Cardiology (ESC) guidelines provide recommendations on methods to improve adherence to physicians’ recommendations, and adherence management is also becoming part of care pathways (PriceWaterhouseCoopers, 2007; Mancia et al., 2013; Heidbuchel et al., 2015); however, more global guidance that is not disease specific is needed. Furthermore, the guidelines should be more prescriptive and less generic.

Drug adherence problems are characterized by two major patterns: non-persistence and good persistence but poor implementation of the dosing regimen (primarily missed doses and drug holidays). Identification of the problem is crucial as the prevention strategy depends on the type of pattern. Suboptimal implementation may lead to poor blood pressure control, which in turn can lead to non-persistence (Blaschke et al., 2012).

In addition to determining whether drugs are taken, it is important to assess drug adherence. The difficulty of accurately assessing adherence is highlighted by a study by Meddings et al. (2012) where primary care providers recognized non-adherence for less than half of those patients who had significant gaps in their refill history. Apps are a conceptual way to implement adherence; however, there are too many, they work for a limited time, are often generic and even if they provide feedback to the healthcare provider they are too complicated. Adherence data is needed at the point of care.

**FIGURE 4:**
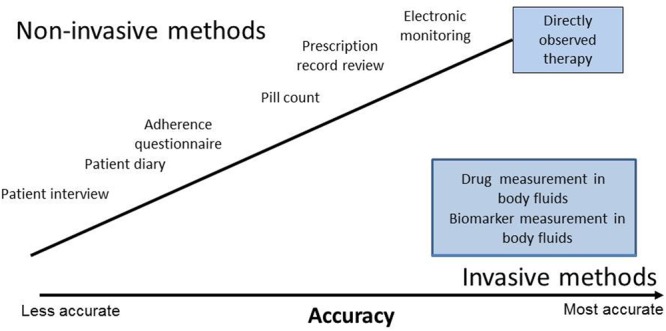
**Non-invasive and invasive methods of measuring adherence**.

There are several non-invasive and invasive methods of measuring adherence (**Figure 4**). There is no one gold standard method of measuring adherence; a combination of methods should be used to measure initiation, implementation and persistence which should be individualized (**Figure 1**; Gupta et al., 2010). The most accurate methods are electronic monitoring and drug measurement. Electronic monitoring consists of automatic compilation of drug dosing history data that may be useful in the management of patients with resistant hypertension (Burnier et al., 2001). The Medication Event Monitoring System (MEMS^®^) is an example of electronic monitoring of adherence that records the date and time when the package is opened to remove medication. Although not available in all countries, they are recognized as an underutilized resource. They have the advantage of being a dynamic measure, but do not prove ingestion. Monitoring of drug levels has been shown to improve blood pressure control at follow-up visits (Brinker et al., 2014). Whilst blood or urine drug measurements prove ingestion they are invasive, costly, and are very limited as they do not reflect the behavior of medication taking. The Medication Event Monitoring System (MEMS) is an example of an electronic medication monitoring, measurement and adherence system. A meta-analysis of the impact of different strategies to improve adherence and blood pressure control found that collaboration with healthcare partners has the greatest impact (Glynn et al., 2010).

Other systems in development for monitoring adherence include ingestible sensor systems combined with wireless observed therapy (Belknap et al., 2013) and electronically chipped packaging. It is anticipated that in the future, adherence monitoring will become routine for chronic conditions at specific time points, i.e., initiation and treatment failure.

## Strategies to Improve Adherence

A number of different strategies can be used to improve blood pressure control (**Table 1**; Volpe et al., 2012a,b, 2013). Patients’ preferences as to methods of improving adherence remain an “unknown, unknown.” The majority of patients need combination therapy to achieve blood pressure control; however, pill burden is associated with lower adherence (Gerbino and Shoheiber, 2007). Dosing frequency is important and can in cases of less frequent dosing lead to non-intentional non-persistence. Treatment simplification is one of the most straightforward ways to enhance adherence, by facilitating implementation of the dosing regimen (Redon et al., 2008; Burnier et al., 2009). Single-pill FDCs can reduce pill burden and simplify treatment regimens (Mancia et al., 2013). FDCs significantly improve adherence and improve BP normalization ratios compared with free combinations (Gupta et al., 2010; Sherrill et al., 2011). Efforts to take advantage of the benefits of FDCs for improving adherence include an angiotensin-receptor-blocker-based hypertension treatment platform. This is a practical tool which has been devised to guide the use of single-pill FDCs containing two- and even three drugs in clinical situations commonly seen in hypertension (Volpe et al., 2014). FDCs can be expensive in some countries and may constitute a barrier for adherence.

**Table 1:** Strategies for improving blood pressure control (Volpe et al., 2012a,b, 2013).

● Define and share key therapeutic targets
● Prepare Consensus Document and Practical Guidelines, share with General Medicine
● Interventions for information and motivation among the population (blood pressure control, virtuous lifestyle, adherence to prescribed treatment, use of mass media and social networks)
● Promotion of the use of check-lists, database, clinical case records and network of dedicated outpatient care units
● Dialog with stake-holders
● Promote long-lasting anti-hypertensive drugs in mono and combination therapy
● Promote therapeutic simplification

Patients’ awareness of their adherence patterns can change their behavior (Vrijens et al., 2006). The key elements to changing patients’ behavior include: education, motivation, and measurement (Vrijens et al., 2014). Packaging is an underused opportunity to effectively manage medication adherence. It has a role to play in measurement and provision of information. The ESH/ESC guidelines include reminder packaging as a method of improving adherence to physicians’ recommendations (Mancia et al., 2013). A real-world assessment of the impact of reminder packaging in the US has shown that it can improve rates of adherence and persistence to antihypertensive treatment (Dupclay et al., 2012). A higher proportion of patients who received their prescribed medication in reminder packaging remained on treatment and were less likely to discontinue therapy compared with the non-reminder packaging group. This approach to improving adherence through improvements in packaging is now being applied within Europe. Recently, Daiichi Sankyo re-designed its hypertension medication packaging to include the following features: top-opening to provide easy access to medication, improve convenience, and hopefully lead to patients keeping the packaging; an intake reminder inside the box to reduce the risk of missing pills (it is important to link an activity to the same time every day, to facilitate patient engagement); instant weekday visibility, makes patients’ aware if they have missed a dose; digital patient product information available via a QR-code provides access to relevant information in an easy to read, legible format. Finally, a blister reminder helps to prevent patients from running out of medication.

Re-packing products in this way might be considered as a major step in improving initiation, supporting implementation and ultimately persistence to treatment. Other important considerations to engage discussion between patients and health care providers are: materials to support counseling; dummy packaging. It is recognized that pharmaceutical manufacturers could do more with regards to improving packaging of medications; small changes may have a meaningful impact on adherence.

## Conclusion

The advent of uniquely powerful medicines and reliable means to measure adherence highlights the importance of patient adherence, particularly in hypertension. Patient-tailored and measurement-guided interventions are required to achieve sufficient adherence to therapeutic drug regimens. Achieving satisfactory adherence may have far greater impact than any other maneuver to improve antihypertensive treatments, and healthcare systems must evolve to meet this challenge.

## Author Contributions

We confirm that all authors made substantial contributions to the concept and to the drafting of the manuscript or revising it critically for important intellectual content. In additional, all authors provided final approval of the manuscript.

## Conflict of Interest Statement

The authors declare that the research was conducted in the absence of any commercial or financial relationships that could be construed as a potential conflict of interest.
